# Assessing the prevalence of neutralizing antibodies (NAbs) to SARS-CoV-2 during three years of the COVID-19 pandemic

**DOI:** 10.1016/j.bbrep.2024.101903

**Published:** 2024-12-17

**Authors:** Marina dos Santos Barreto, Ronaldy Santana Santos, Eloia Emanuelly Dias Silva, Deise Maria Rego Rodrigues Silva, Pedro Henrique Macedo Moura, Pamela Chaves de Jesus, Jessiane Bispo de Souza, Lucas Alves da Mota Santana, Adriana Gibara Guimarães, Lysandro Pinto Borges

**Affiliations:** a Department of Pharmacy, Federal University of Sergipe, São Cristóvão, 49100-000, SE, Brazil; b Department of Biology, Federal University of Sergipe, São Cristóvão, 49100-000, SE, Brazil; c Graduate Program in Dentistry, Federal University of Sergipe, São Cristóvão, 49100-000, SE, Brazil; d Department of Immunology, Institute of Biomedical Sciences, University of São Paulo, São Paulo, 05508-000, SP, Brazil

**Keywords:** COVID-19, Pandemic, Vaccine, Neutralizing antibodies, Brazil

## Abstract

The development of COVID-19 vaccines has been an important step in the fight against the pandemic. However, it is still necessary to understand the influence of factors that can alter the immune response. In general, doses need to be updated frequently, and care must be taken to control the virus that is still circulating worldwide. In this study, we evaluated the neutralizing antibodies (NAbs) against SARS-CoV-2 in northeast Brazil. The study was divided into three phases (T1, T2, and T3) and included 297 participants. The three phases occurred in three different years of the pandemic (2021, 2022 and 2023). We obtained higher mean NAbs in T2 and T3 when most of the participants had already completed their vaccination program. No significant difference in the distribution of NAbs and sex was observed (p > 0.05). It was shown that there is a difference in the expression of NAbs in the different amounts of doses, with individuals with no dose obtaining a significantly lower average than those who took the vaccine. Regarding age, the average NAbs were higher with increasing age. In this study, we assess the prevalence of NAbs in three pandemic phases, making it possible to understand the importance of vaccine updating in maintaining immunity.

## Highlights

• Not vaccinated individuals showed significantly lower NAbs averages than those who received vaccine doses.
• A positive and significant correlation was observed between the number of doses and the production of NAbs.
• The average NAbs levels increased with age across all three study phases.

## 1 Introduction

The COVID-19 pandemic has affected millions of people worldwide. The arrival of vaccines created a safer environment was built for everyone, making it possible to minimize protective measures and significantly reducing the number of cases and deaths. In Brazil, four vaccines have been made available to the population: CoronaVac, ChAdOx1 (AstraZeneca/Oxford), BNT162b2 (Pfizer/BioNTech), and Ad26.COV2.S (Janssen-Cilag) [1]. However, while the impact of the virus has been significantly minimized, there are still people who become infected and develop more severe forms of the disease [1]. Furthermore, vaccine adherence has become an issue of concern worldwide, with not all populations complying with the World Health Organization (WHO) recommended vaccination schedule and keeping it up to date [2]. In Brazil, just over 50 % of the population received the booster dose [3], reflecting the scenario of non-compliance with the vaccine update recommendation. Vaccine adherence continues to be an important issue. Recently, Brazil has seen the resurgence of diseases previously considered eradicated, such as rubella and measles [4].

The latest vaccine update is the bivalent vaccine, which was developed for greater effectiveness against SARS-CoV-2 variants, that can easily evade the immune system. However, according to data published in December 2023, only 17 % of Brazilian adults have taken this vaccine. In Sergipe, a state in northeastern Brazil, data from January 2024 show that just over half the population had received the booster dose [1]. Concerned about compliance with the vaccination update and the immune status against SARS-CoV-2, we assessed the presence of neutralizing antibodies (NAbs) against SARS-CoV-2 in the population of a state in northeastern Brazil.

## 2 Material and methods

The study was approved by the National Ethics Committee (under CAAE 31018520.0.0000.5546, September 2020) and was conducted according to the Declaration of Helsinki. Informed consent was obtained from all study participants. This cross-sectional study consisted of three phases, carried out during the first years of the COVID-19 pandemic in a Brazilian state. A total of 297 individuals participated in the study. Inclusion criteria were age 40 years or older, not having been diagnosed with a chronic or terminal disease, not having received a positive diagnosis for COVID-19, and consenting to participate in the study. Participants were asked if they had ever had diagnostic tests for COVID-19 ( RT-PCR and antigenic test). If so, they were asked to provide the results of the report. Those who hadn't been tested before confirmed that they had never been in direct contact with someone who was positive or felt any kind of flu-like symptoms, complying strictly following the pandemic recommendations. The first test (T1) was carried out in July 2021 (*n* = 189), the second test (T2) in July 2022 (*n* = 58) and the third in July 2023 (*n* = 50). The reduction in the number of participants was due to the difficulty in fitting the inclusion criteria, especially not having had COVID-19 previously.

First, participants completed a questionnaire that included information about their sex, age, diagnosed health problems, the doses and brands of vaccines used, and whether they had received a homologous or heterologous vaccination. Homologous vaccination refers to individuals who have only used only one type of vaccine, and heterologous vaccination refers to individuals who have used different types of vaccines in their vaccination schedule. After this stage, we collect the blood in a tube containing a separation gel and store it under appropriate conditions and temperatures (2-8 °C) for later laboratory analysis of neutralizing antibodies (NAbs) against SARS-CoV-2. For this, we used the ichroma™ COVID-19 nAb device (https://www.boditech.co.kr/en/support/id/226). ichroma COVID-19 nAb is a fluorescence immunoassay (FIA) that evaluates the interference of antibodies with a possible neutralizing function against SARS-CoV-2 in the interaction between the receptor binding domain (RBD) and the surface receptor angiotensin-converting enzyme 2 (ACE-2). The test has a sensitivity of 95.8 % and a specificity of 97 %. After reading the sample, the device provides a quantitative result, with results ≥30 % being reactive and results <30 % being non-reactive for the NAbs.

Statistical analysis was performed using IBM® SPSS® software (version 26.0 for Windows). The Kruskal-Wallis test for independent samples was used to assess whether the mean NAbs were evenly distributed between testes. In addition, Spearman's correlation test was used to evaluate the NAbs and the amount of dose and age of the participants. Results were considered significant if *p* < 0.05 and non-significant when *p* > 0.05. We used jamovi® (https://www.jamovi.org/) to graphically display the distribution of NAbs in the different vaccine doses used by the study participants.

## 3 Results

A total of 297 people took part in the study, 80 (26.9 %) female and 217 (73.1 %) male. The average age was 55.75 years (*SD* = 14.07; CI95 % = 54.15–57.36). At T1, the participants had been vaccinated with none, one or two doses. At T2, the current vaccination schedule was two and three doses. In T3, some participants had used between two and five doses. When comparing the distribution of NAbs in the three tests, the Kruskal-Wallis test provided a statistically significant difference (*p* < 0.01), with the means for the second (*m* *=* 88.54 ± 17.91) and third (*m* *=* 80.58 ± 30.85) tests being higher than the first (*m* *=* 46.98 ± 34.54).

In the three phases, the mean NAbs for men was higher than for female. At T1 the mean for men was 47.46 ± 34.73, and for females, 44.30 ± 47.45. At T2, the mean for males was 91.93 ± 12.43, and 84.91 ± 22.01 for females. At T3, a higher NAbs mean was also observed for men (male *m* *=* 86.20 ± 25.97 *vs.* female *m* = 73.97 ± 35.19). This may be due to menopause, as lower levels of estradiol affect the immune system, and in T3 there were more elderly people [5]. However, there was no statistically significant relationship between sex and the NAbs levels (*p* > 0.05).

When applying Spearman's correlation test, we obtained a significant correlation between the production of NAbs and the age of the participants (*r* = 0.274, *p* < 0.01), suggesting that the older the participant, the greater the production of antibodies. In addition, a correlation was found between age and the number of doses used by the participants (*r* = 0.637, *p* < 0.01). Furthermore, a positive and statistically significant correlation was observed between the number of doses and the production of NAbs (r = 0.441; p < 0.01). Therefore, the correlation between age and the production of NAbs may have been due to the majority of elderly people in T3, where more doses of the vaccines were available.

**Fig. 1:**
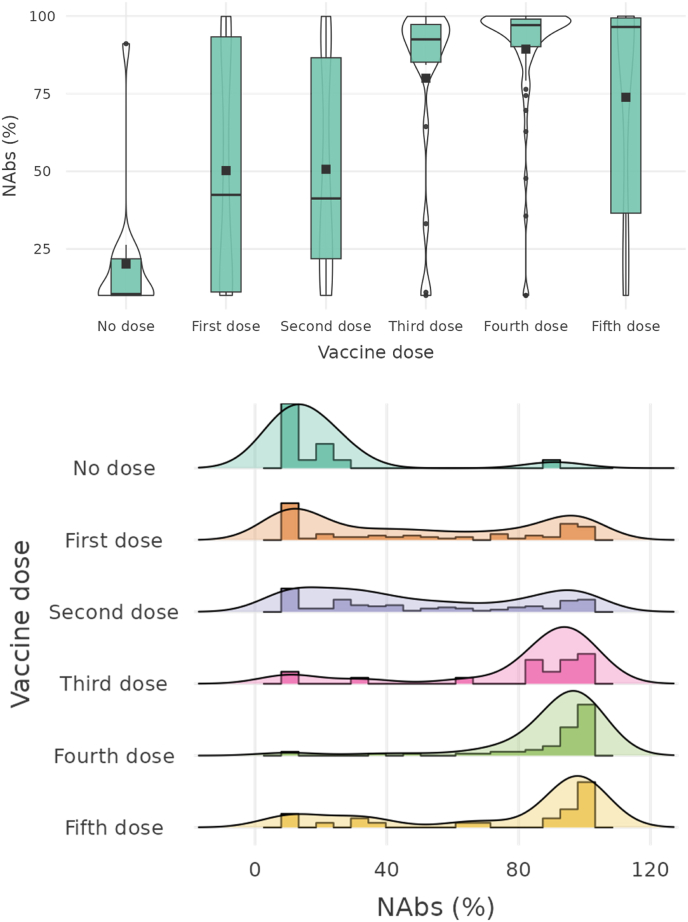
Distribution of anti-SARS-CoV-2 NAbs (%) for the different quantities of vaccine doses used. No dose (*n* = 14; *m* = 20.11 ± 21.30), First dose (*n* = 77; *m* = 50.22 ± 36.44), Second dose (*n* = 102; *m* = 50.65 ± 33.49), Third dose (*n* = 19, *m* = 79.98 ± 29.07), Fourth dose (*n* = 60; *m* = 89.36 ± 19.36), Fifth dose (*n* = 25; *m* = 73.85 ± 35.16). The dots on the graphs represent the outliers. The black squares represent the mean for each group and the black line indicates the median.

The Kruskal-Wallis test was applied to assess the distribution of antibodies for the number of doses used and the types of vaccine. A difference in the distribution of NAbs across doses was obtained (*p* < 0.01). It can be seen that the average NAbs for those who had not had the vaccine was below the cut-off index (30%). From the first dose onwards, these antibodies tended to increase to a detectable level , with the highest median levels of NAbs detected ate the third, fourth, and fifth doses. The distribution of mean values for the groups of participants with different doses is shown in Fig. 1.

The distribution of antibodies was analyzed among three groups: individuals who had not received any vaccinations, those who had received a homologous vaccine, and those who had received a heterologous vaccine. The mean NAb level in the non-vaccinated group was found to be 18.67 ± 8.20. The mean NAb level in the homologous vaccine group (36.87 ± 33.14) was found to be lower than that observed in the heterologous vaccine group (56.64 ± 38.98). A Kruskal-Wallis test was used to determine if there were significant differences between the groups, and the results indicated that there were statistically significant differences (p < 0.05).

## 4 Discussion

This study assessed the prevalence of neutralizing antibodies against COVID-19 in the population of a Brazilian state during three years of the pandemic. We found a greater presence of these antibodies in the last two tests, where the population had already completed the vaccination schedule for the most part, compared to the first test. This result may be a reflection of the progress and adherence to the vaccination process in Brazil, since we also found a difference in the mean NAbs for the group that used any doses of the vaccine, compared to those who completed the vaccine schedule or used at least one booster dose (two to five dose group). The genetic factors of each individual may also explain the variability in the response of the population that used the same number of vaccine doses [6]. This suggests the importance of updating the vaccine to maintain the immune system against the COVID-19 virus, even in a more attenuated phase of the pandemic [7]. Previous studies have reported the importance of updating the vaccination schedule and adhering to the bivalent vaccine to continue immunizing against the coronavirus. This makes it possible to combat the virus and minimize the chances of a new pandemic wave appearing [8,9].

Updating the vaccination schedule is important to minimize the impact of the new SARS-CoV-2 variants, such as Omicron and its subvariants [1]. The bivalent vaccine was available in Brazil in February 2023 and had a positive effect on neutralizing antibody production in our study, given the average of antibodies in T3. However, the decrease in participant numbers in T2 and T3 could limit the generalizability of our findings. It is worth noting that in the T3, there was a higher prevalence among the elderly, showing the possible effect of the bivalent vaccine on this population, which suffers from immunosenescence [10], affecting the immune system and, consequently, the production of antibodies to defend against the coronavirus. This may have been one of the main causes for the drop in NAbs levels on the third test. A study carried out in Japan demonstrated the importance of the bivalent vaccine in the population aged 65 and over [11].

This study has some limitations. These include the reduction in the population in T2 and T3, mainly due to the difficulty in keeping the population within the inclusion criteria. In addition, we could not say whether all the participants had never been infected, since they were not monitored regularly for viral testing.

With this study, it was possible to observe the behavior of a state's population at three different times during the pandemic. It was found that individuals with fewer doses or an incomplete vaccination schedule generate a lower production of NAbs compared to the population that used the booster dose. In addition, we emphasize the importance of continuing studies that assess the immunity of individuals in general, especially the elderly.

## Ethics in publishing

The study was conducted following the Declaration of Helsinki, and approved by the Ethics Committee of Research in Brazil (Comitê de Ética em Pesquisa (CEP) platform) under the protocol code 31018520.0.0000.5546 in September 2020. All participants gave their formal consent to take part in this study.

## CRediT authorship contribution statement

**Marina dos Santos Barreto:** Writing – original draft, Methodology, Investigation, Data curation, Conceptualization. **Ronaldy Santana Santos:** Writing – original draft, Methodology, Investigation, Data curation. **Eloia Emanuelly Dias Silva:** Writing – original draft, Methodology, Investigation, Data curation. **Deise Maria Rego Rodrigues Silva:** Writing – original draft, Methodology, Investigation, Data curation. **Pedro Henrique Macedo Moura:** Writing – original draft, Methodology, Investigation, Data curation. **Pamela Chaves de Jesus:** Writing – original draft, Methodology, Investigation, Data curation. **Jessiane Bispo de Souza:** Writing – original draft, Methodology, Investigation, Data curation. **Lucas Alves da Mota Santana:** Writing – review & editing, Data curation, Conceptualization. **Adriana Gibara Guimarães:** Writing – review & editing, Methodology, Conceptualization. **Lysandro Pinto Borges:** Writing – review & editing, Project administration, Investigation, Conceptualization.

## Funding sources

The study received no funding.

## Declaration of competing interest

The authors declare that they have no known competing financial interests or personal relationships that could have appeared to influence the work reported in this paper.

## Data Availability

Data will be made available on request.

## Abbreviations

Nabs -: Neutralizing Antibodies
T1 -: First test
T2 -: Second test
T3 -: Third test
